# UMG Lenti: Novel Lentiviral Vectors for Efficient Transgene- and Reporter Gene Expression in Human Early Hematopoietic Progenitors

**DOI:** 10.1371/journal.pone.0114795

**Published:** 2014-12-12

**Authors:** Emanuela Chiarella, Giovanna Carrà, Stefania Scicchitano, Bruna Codispoti, Tiziana Mega, Michela Lupia, Daniela Pelaggi, Maria G. Marafioti, Annamaria Aloisio, Marco Giordano, Giovanna Nappo, Cristina B. Spoleti, Teresa Grillone, Emilia D. Giovannone, Raffaella Spina, Francesca Bernaudo, Malcolm A. S. Moore, Heather M. Bond, Maria Mesuraca, Giovanni Morrone

**Affiliations:** 1 Laboratory of Molecular Haematopoiesis and Stem Cell Biology, Dept. of Experimental and Clinical Medicine, University of Catanzaro Magna Græcia, 88100, Catanzaro, Italy; 2 Laboratory of Molecular Oncology, Dept. of Experimental and Clinical Medicine, University of Catanzaro Magna Græcia, 88100, Catanzaro, Italy; 3 Dept. of Cell Biology, Memorial Sloan-Kettering Cancer Center, New York, New York, 10065, United States of America; The University of Tennessee Health Science Center, United States of America

## Abstract

Lentiviral vectors are widely used to investigate the biological properties of regulatory proteins and/or of leukaemia-associated oncogenes by stably enforcing their expression in hematopoietic stem and progenitor cells. In these studies it is critical to be able to monitor and/or sort the infected cells, typically via fluorescent proteins encoded by the modified viral genome. The most popular strategy to ensure co-expression of transgene and reporter gene is to insert between these cDNAs an IRES element, thus generating bi-cistronic mRNAs whose transcription is driven by a single promoter. However, while the product of the gene located upstream of the IRES is generally abundantly expressed, the translation of the downstream cDNA (typically encoding the reporter protein) is often inconsistent, which hinders the detection and the isolation of transduced cells. To overcome these limitations, we developed novel lentiviral dual-promoter vectors (named UMG-LV5 and –LV6) where transgene expression is driven by the potent UBC promoter and that of the reporter protein, EGFP, by the minimal regulatory element of the WASP gene. These vectors, harboring two distinct transgenes, were tested in a variety of human haematopoietic cell lines as well as in primary human CD34^+^ cells in comparison with the FUIGW vector that contains the expression cassette UBC-transgene-IRES-EGFP. In these experiments both UMG-LV5 and UMG–LV6 yielded moderately lower transgene expression than FUIGW, but dramatically higher levels of EGFP, thereby allowing the easy distinction between transduced and non-transduced cells. An additional construct was produced, in which the cDNA encoding the reporter protein is upstream, and the transgene downstream of the IRES sequence. This vector, named UMG-LV11, proved able to promote abundant expression of both transgene product and EGFP in all cells tested. The UMG-LVs represent therefore useful vectors for gene transfer-based studies in hematopoietic stem and progenitor cells, as well as in non-hematopoietic cells.

## Introduction

Gene transfer-based strategies represent a valuable asset in the characterization of hematopoietic regulators and in the identification and dissection of the oncogenic potential of a variety of leukemia-associated candidate oncogenes. Hematopoietic malignancies, and in particular acute myeloid leukemias (AMLs), are derived from the accumulation of progenitor cells arrested at early stages of differentiation and are characterized by the presence of non-random genetic aberrations that include gross chromosomal abnormalities and more subtle mutations affecting key regulatory genes. In the past few years, a wealth of studies have demonstrated that enforced expression of such aberrant genes in stem and progenitor cells of the hematopoietic system can confer a strong proliferative advantage on these cells, resulting in their selective expansion in vitro (and in some cases in vivo), and can interfere to different degrees with their normal differentiation [1]–[11]. Gamma-retroviral and HIV-1-derived lentiviral vectors are the most commonly-used vehicles for such gene transfer-based studies, owing to their ability to accommodate relatively large fragments of exogenous DNA, as well as to their efficiency in transducing hematopoietic stem and progenitor cells (HSPCs) and integrating stably in the genome of the infected cells, thus promoting constitutive expression of the transgenes. Lentiviral vectors have gained particular favour because they can efficiently infect quiescent or slowly-dividing cells, which makes them particularly well-suited for the transduction of the most primitive hematopoietic progenitors [12]–[13].

In these studies, the possibility to monitor the subset of cells infected by the viral vectors (and hence expressing the relevant transgenes) is of paramount importance. The relative expansion of these cells within the total cell population will indicate that the expression of the protein(s) studied results in selective growth/self-renewal advantage compared to the non-infected counterpart [2]–[6]. Moreover, the ability to isolate the transduced cells is advantageous and often essential, because it yields homogeneous populations of transgene-expressing cells for more sophisticated biochemical and functional analyses, as well as gene expression profiling for the discovery of downstream targets of the proteins of interest [2], [4]–[7], [11]. For these purposes, it is crucial to achieve stable co-expression in the target cells of the transgenes and of reporter genes that encode proteins whose presence can be detected by flow cytometry (proteins instrinsically fluorescent [2]–[11] or cell surface-associated molecules that are recognized by specific, fluorophore-conjugated, antibodies or ligands [10]). To ensure the simultaneous expression of transgenes and reporter genes, the most common approach is based on the insertion between their cDNAs of virus-derived intra-ribosomal entry site (IRES) elements, thus generating bi- or poly-cistronic mRNAs under the transcriptional control of a single promoter [14]. In these constructs the cDNA encoding the protein of interest is typically located upstream of the IRES, and the reporter gene is downstream. While these vectors generally promote the expression of acceptable levels of transgene products and of reporter proteins in the majority of cell lines, the efficiency of the IRES sequence - particularly in the context of the lentiviral genome - is frequently inconsistent in primary hematopoietic cells. This results in poor translation of the downstream coding sequence, and therefore in low levels of reporter protein [15] that render the identification and/or isolation of the infected cells problematic. To circumvent this limitation we have produced, and describe here, novel IRES-containing or dual promoter-based lentiviral vectors containing the potent Ubiquitin-C gene promoter and the regulatory element of the Wiskott-Aldrich syndrome gene, that have proven capable of inducing the abundant expression of both transgene and reporter gene in a variety of human hematopoietic cell lines with diverse phenotypes and, more importantly, in primary human early hematopoietic progenitors. These vectors represent potentially valuable tools for gene transfer-based studies in hematopoietic stem and progenitor cells.

## Materials and Methods

### Ethical statement

The lentiviral transductions of primary human cells were approved by the Institutional Review Board (Comitato Etico Azienda Ospedaliera Mater Domini) on 18 September 2009. The only primary human samples used in this study were commercially-available human purified CD34^+^ cells (Lonza). Informed consent was therefore not applicable.

### Cell lines and culture conditions

The human hematopoietic cell lines K562 (ATCC-CCL-243), HL-60 (ATCC-CCL-240), MV4;11 (ATCC-CRL-9591), THP-1 (ATCC-TIB-202), Jurkat (ATCC-TIB-152) and DeFew [16] were cultured in RPMI 1640 medium. The non-hematopoietic cell lines, DAOY (human medulloblastoma - ATCC-HTB-186), HEK293T (human embryonic kidney - ATCC-CRL-3216), MS-5 (murine, stromal - DSMZ-ACC 441) and NIH-3T3 (mouse embryonic fibroblast - ATCC-CRL-1658) were cultured in Dulbecco's modified Eagle medium (DMEM). Tissue culture media were supplemented with 10% fetal bovine serum (FBS), 50 U/ml of penicillin and 50 µg/ml streptomycin and glutamine (glutamax). All tissue culture reagents were from Life Technologies (Milano, ITALY).

Cord blood-derived early hematopoietic progenitors (>95% CD34^+^) were purchased from Lonza and cultured at 1×10^5^ cells/ml for transfection and at 1×10^4^ cells/ml for determination of growth, at 37°C in 5% CO_2_ in HPGM medium (Lonza, ITALY) supplemented with 100 ng/ml of Stem Cell Factor (SCF), FLT3 Ligand (FL) and Thrombopoietin (TPO) (PeproTech, UK). For colony-forming cell (CFC) assays, 500 cells were plated in triplicate in 0.5 ml of methylcellulose-containing medium (Methocult H4230, Stem Cell Technologies) supplemented with 20 ng/ml interleukin-3, interleukin-6, stem cell factor, granulocyte colony-stimulating factor, FLT-3 ligand, and 1 U/ml Epo (all from PeproTech, UK). Colonies were scored after 2 weeks.

### Construction of multigene lentiviral vectors

The lentiviral IRES-containing vectors FUIGW and FUIGW-ZNF521 have been previously described [17]. To construct the dual promoter vectors UMG-LV5 and UMG-LV6, the synthetic oligonucleotide containing a polyA signal derived from that of the human growth hormone, as well as multiple cloning sites (MCS), was inserted in the FUIGW vector backbone. (ΔPacI-polyA signal-EcoRI- PacI- BamHI- ΔEcoRI: CAATTCCTCATTTTATTAGGAAAGGACAGTGGGAGGAATTCTTAATTAAGGATCCA).

The UMG-LV5 plasmid was constructed by inserting an expression cassette containing the 170 bp WASP promoter (W) and the EGFP coding sequence in PacI-EcoRI upstream of the polyA signal. The fragment UBC promoter-MCS (PacI-BamHI) from FUIGW was cloned into the PacI and BamHI cloning sites.

To construct the UMG-LV6 plasmid, a synthetic adapter (ΔEcoRI–BamHI-PacI-NotI-ΔBamHI: AATTAGGGATCCGTTAATTAAGGCGGCCGCTA) was inserted in the modified version of FUIGW containing the polyA signal described above. The WASP promoter-EGFP expression cassette was cloned into the PacI-NotI sites and the NotI site was later deleted. A PacI-BamHI fragment from FUIGW encompassing the UBC promoter and the multiple cloning sites (MCS) (where XbaI, EcoRI and BamHI are the only unique cloning sites) was then cloned in antisense orientation, utilizing the PacI and BamHI cloning sites of the adapter, upstream of the polyA signal.

The lentiviral vector UMG-LV11 was prepared by modifying the FUIGW vector by the addition of the EGFP-IRES-MCS expression cassette downstream of UBC promoter. Briefly, to construct the pUMG-LV11 plasmid, an adapter sequence containing multiple cloning restriction sites (AgeI-NotI-XbaI-NheI-BamHI-EcoRI) was inserted downstream of the UBC promoter in the FUIGW backbone, replacing the MCS-IRES-EGFP element. The EGFP gene from pFUGW was then cloned into the AgeI-NotI sites of the adapter. The IRES sequence from pWZL was amplified using primers complementary to the 5′ and 3′ sequences, with additional sites for NotI and XbaI respectively. The PCR product was digested with these enzymes, separated on a 1% agarose gel, purified by QIAEX II Gel Extraction Kit (QIAGEN) following the manufacturer's instructions, and then cloned NotI-XbaI in the MCS to generate pUMG-LV11.

The pCDH-CMV-EF1α-copGFP plasmid was purchased from Systems Biology (Mountain View, CA, USA); the pHIV- EF1α-IRES-EGFP was obtained from Addgene (http://www.addgene.org/).

The cDNA for 3xFLAG-ZNF521 and for 3xFLAG-MSI2 were subcloned as XbaI-BamHI fragments in the corresponding restriction sites of the relevant lentiviral plasmids; the MLL-AF9 cDNA, kindly provided by Prof. E. Canaani (Weizmann Institute of Science, Rehovot, Israel), was subcloned as an EcoRI-EcoRI fragment in the EcoRI site of pUMG-LV6.

### Lentivectors production and cell transduction

Viral stocks were produced in HEK293T cells (1×10^7^) by co-transfecting 10 µg of each multigene transfer vector plasmids with 10 µg of packaging plasmid pCMV-ΔR8–91 and 2 µg of pCMV-VSVG, as previously described [18]. Six hours after transfection the medium was changed with RPMI or DMEM supplemented with 3% FBS, according to target cell growth conditions. For the transduction of CD34^+^ cells, serum-free HPGM medium was used. After 24 h and 48 h, lentivirus-containing supernatants were collected, centrifuged at 400× g for 5 min at 4°C to remove floating cells and debris, filtered through 0.45 µm filters (Millipore) and used to infect target cells.

The titers of lentiviral particles in the supernatants were calculated by transduction of K562 cells with serial dilutions of the supernatants followed by flow-cytometric analysis of EGFP positive cells. In each round of the transduction experiments described in this paper, a multiplicity of infection (MOI) of 2 was used.

Transduction was enhanced by spin-inoculation of the cultures. Cells were seeded at 2×10^5^ cells/well in 12-well-plate and 2 ml of viral supernatant were added to each well in the presence of 8 µg/ml polybrene and 10% FBS; cells were centrifuged at 425× g for 50 min at 32°C. Next day, fresh viral supernatants were added to the cells followed by another spin-inoculation. Virus-containing supernatants were completely removed after 48 hours and cells cultured for an additional 5 days before FACS and Western blotting analysis.

### Flow cytometric analysis and sorting

To evaluate the transduction efficiency, EGFP positive cells were detected using a FACScan flow cytometer (Beckton-Dickinson). Analysis was performed with FlowJo software. Untransduced cells were used as controls. Sorting of transduced K562 cells was performed using the BD FACSAria III. The sorted populations were analyzed by flow cytometry to confirm their purity.

### Protein extracts and Western blot analysis

Cell pellets were resuspended in hypotonic lysis buffer (10 mM Hepes pH 7.9, 10 mM KCl, 0.1 mM EDTA pH 8.0, protease inhibitors (P8849, Sigma) and phosphatase inhibitor cocktails 2 and 3 (P0044, P5726 Sigma) and incubated on ice for 20 minutes. After the addition of 0.25% Igepal-630 (NP40), samples were centrifuged at 1200× g for 5 minutes: the supernatants (containing the cytosolic extracts) were recovered. Nuclear pellets were resuspended in nuclear extract buffer (20 mM Hepes pH 7.9, 0.4 M NaCl, 1 mM EDTA pH 8.0, protease and phosphatase inhibitors. The lysates were subjected to three rounds of alternating vortex mixing and ice-cooling, and then were centrifuged at 15300× g for 20 minutes and the supernatants (nuclear extracts) were collected.

For total protein extraction, cultured cells were resuspended in lysis buffer (250 mM Tris-HCl pH 7.5), and then subjected to three cycles of freezing and thawing (−70/+37°C). The lysate was centrifuged at 15300× g for 20 minutes and the supernatants (whole-cell extracts) recovered. The protein concentration was determined using the Bio-Rad Assay Reagent.

30 µg of protein extracts were denatured, reduced, separated by electrophoresis on 4–12% NuPAGE Novex bis-Tris gradient polyacrylamide gels (Life Technologies, ITALY) and electrophoretically transferred onto nitrocellulose filters. The membranes were then quenched with 5% blocking solution (BioRad) and incubated with primary and then, when applicable, with secondary antibodies. The HRP-conjugated anti-FLAG monoclonal antibody (M2 A8592, Sigma-Aldrich, ITALY) was used at a 1∶10000 dilution for the detection of FLAG epitope-tagged proteins. Anti-HDAC1 (H3284), anti-actin (A4700) and anti-GFP (N-terminal G1544) (Sigma-Aldrich, ITALY) were used at 1∶12000 and 1∶2000 dilutions respectively and detected with anti rabbit secondary HRP-conjugated antibodies (Santa Cruz, Biotechnology). HRP was revealed using the ImmunoCruz Western blotting luminal reagent (Santa Cruz, Biotechnology) by autoradiography.

The original, full scans of the Western blots shown in this paper are reported in S5 and S6 Figures.

### Quantitative RT-PCR measurement of MLL-AF9 expression in transduced CD34^+^ cells

RNA extraction, cDNA synthesis and Q-RT-PCR were performed as previously described [19] using the following oligonucleotides designed to amplify a cDNA fragment spanning the junction of the MLL and AF9 moieties of the fusion MLL-AF9 mRNA:

FWD: CACCTACTACAGGACCGCCAAG

REV: CTAGGTATGCCTTGTCACATTCACC

For normalization, GADPH was used as described [19].

## Results

### Structure of the UMG-LV5, UMG-LV6 and UMG-LV11 vectors

**Figure 1:**
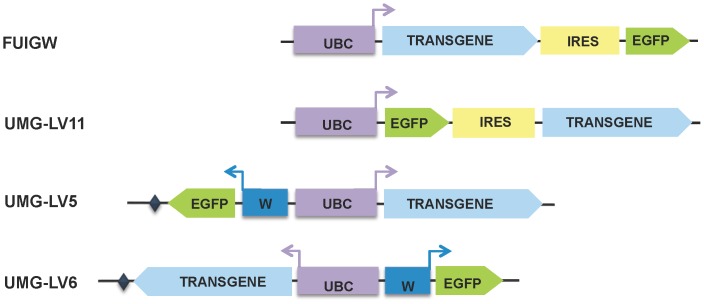
Schematic diagram of IRES-based and dual promoter lentiviral vectors. The expression cassettes of the lentiviruses used in this study are illustrated. The two IRES-containing vectors, FUIGW and UMG-LV11, differ for the position of the transgene and EGFP cDNA relative to the IRES element. In both viruses the transcription of this bicistronic unit is driven by the UBC promoter. The UMG-LV5 and UMG-LV6 vectors use independent promoters positioned back-to-back: UBC for the transgene and WASP (W) for EGFP. These dual-promoter vectors differ only for the orientation of the expression cassette. A short synthetic polyA signal, based on that of the human growth hormone gene, is downstream of the transcriptional unit in anti-sense orientation and is indicated by a diamond (♦).

The structure of the the lentiviral vectors used in this study is schematically depicted in Fig. 1.

The top two diagrams illustrate the structure of the IRES-containing expression cassettes. As it can be seen, in the UMG-LV11 construct the EGFP cDNA is directly downstream of the UBC promoter and upstream of the IRES element of the encephalomyocarditis virus derived from the pWZL vector. The two bottom panels show the structure of the dual-promoter constructs, UMG-LV5 and UMG-LV6. These two vectors differ only for the orientation of the expression cassette: in the former, the WASP minimal regulatory element and the EGFP cDNA are in antisense orientation; downstream of the EGFP coding sequence, a short synthetic polyadenylation signal derived from that of the human growth hormone mRNA was inserted. In UMG-LV6 the UBC-transgene transcriptional unit is in antisense orientation, followed by the polyadenylation signal, whereas the WASP promoter-EGFP cDNA unit is in sense orientation. A more detailed graphic map of the constructs, that includes the indication of the most relevant components and of the unique restriction sites in these plasmids, is shown in S1 Figure (panels A, B and C for pUMG-LV5, pUMG-LV6 and pUMG-LV11 respectively).

### Gene transfer in human hematopoietic cell lines with the dual-promoter lentiviral constructs

In an initial set of experiments, we compared the efficiency of FUIGW, UMG-LV5 and UMG-LV6 in inducing the expression of the reporter protein EGFP and of 3xFLAG-tagged zinc finger protein 521 (ZNF521) in a panel of human hematopoietic cell lines with myeloid (K562, HL-60, MV4;11, THP-1), T-lymphoid (Jurkat) or B-lymphoid (DeFew) phenotype.

**Figure 2:**
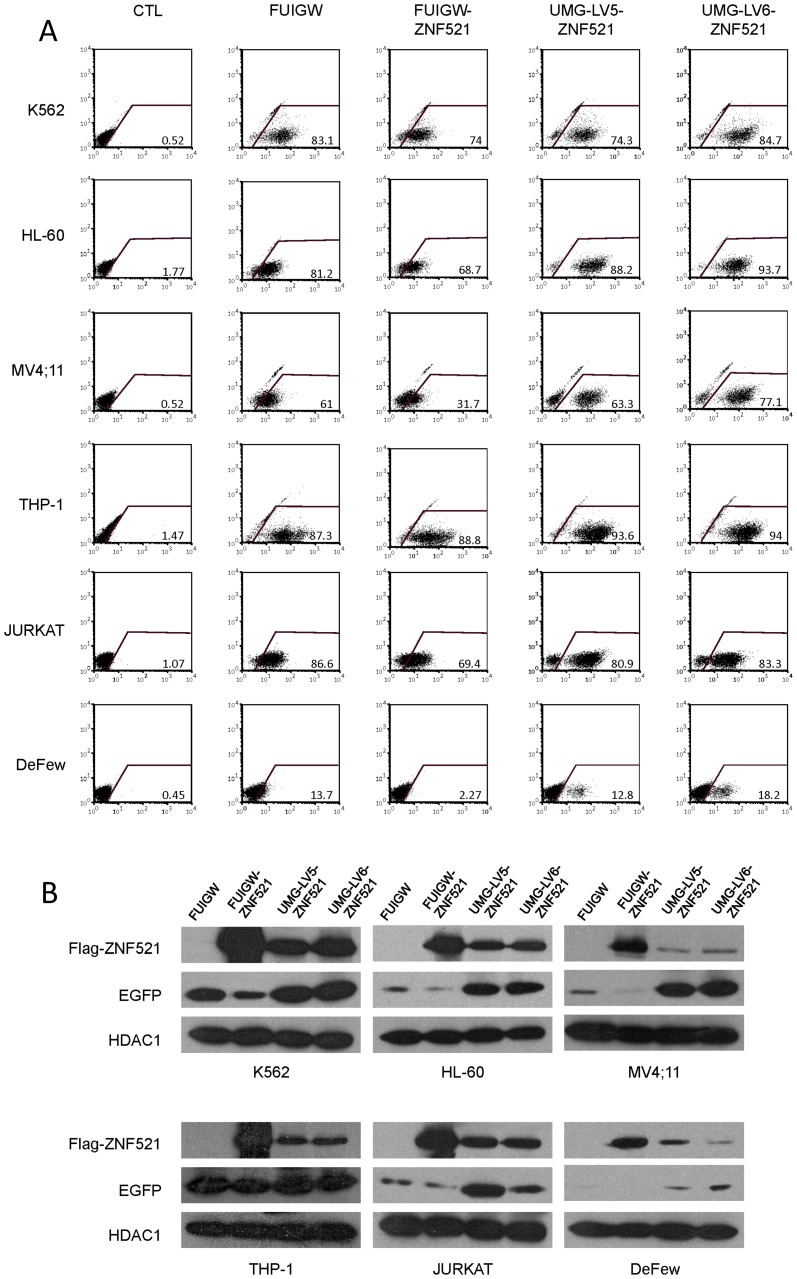
Comparison of the transduction efficiency of FUIGW, UMG-LV5 and UMG-LV6 carrying the ZNF521 cDNA in human hematopoietic cell lines. The cell lines K562, HL-60, MV4;11, THP-1, Jurkat and DeFew were infected as detailed in materials and methods with FUIGW, UMG-LV5 or UMG-LV6 viruses carrying 3xFLAG-ZNF521 cDNA as a transgene and EGFP cDNA as a reporter gene. As a control, void FUIGW vector without transgene cDNA was used. (**A**) Flow-cytometric analysis of EGFP expression in cells exposed to the relevant vectors. The percentages of EGFP-positive cells are indicated. (**B**) Nuclear and cytosolic extracts were prepared as described in materials and methods and analyzed by Western blotting for FLAG-ZNF521 and EGFP expression respectively. HDAC1 was used as a control for the amounts of extract loaded.

ZNF521 is a transcription co-factor that we and others have demonstrated to play a regulatory role in primitive hematopoietic, neural and osteo-adipogenic progenitors [18]–[25]; in addition to its interest as a potential regulator of hematopoiesis, it was selected also because of the large size of its coding sequence (4,080 nt including the sequence encoding the 3xFLAG tag). The target cell lines were transduced as detailed in Material and Methods and then maintained in culture for five days prior to the analyses to avoid artifacts due to pseudotransduction [26]. The expression of EGFP at the single-cell level was measured by flow-cytometry. As shown in panel A of Fig. 2, the majority of the cells in the cultures transduced with each of the viruses used were EGFP-positive, with the sole exceptions of the B-lymphoblastoid cells, DeFew and of the MV4;11 cells transduced with FUIGW-ZNF521. However, a considerable difference in the levels of green fluorescent protein was clearly evident: while both UMG-LV5-ZNF521 and UMG-LV6-ZNF521 induced a strong EGFP expression, that allowed to clearly distinguish EGFP-positive and negative cell subsets, this was not the case in most cultures infected with the IRES-dependent vectors FUIGW or FUIGW-ZNF521. In these samples the EGFP^+^ subpopulation was not as well-defined as in the cultures infected with the dual-promoter lentiviruses. The higher intensity of fluorescence in cells transduced by UMG-LV5-ZNF521 and UMG-LV6-ZNF521 compared to FUIGW-ZNF521 is further highlighted when the EGFP-expressing cells are separately gated based on their “high” or “low” fluorescence level (S2 Figure). The protein levels of both ZNF521 and EGFP were then determined by Western blotting (Fig. 2B). This analysis revealed that, while consistently displaying a lower expression of EGFP, all cell lines infected with FUIGW-ZNF521 produced higher amounts of ZNF521 than those exposed to UMG-LV5-ZNF521 and UMG-LV6-ZNF521.

To corroborate these results using a different transgene, we transduced four of the six cell lines tested in the experiments described in Fig. 2 (ie, K562, HL-60, MV4;11 and Jurkat) with FUIGW, UMG-LV5 and UMG-LV6 carrying the cDNA for 3xFLAG-tagged Musashi 2 (MSI2) instead of ZNF521. MSI2 is an RNA-binding protein [27] that several reports have recently implicated in the maintenance of the immature cell compartment in normal and malignant hematopoiesis [28]–[31], and its coding sequence (987 nt) is considerably shorter than that of ZNF521.

**Figure 3:**
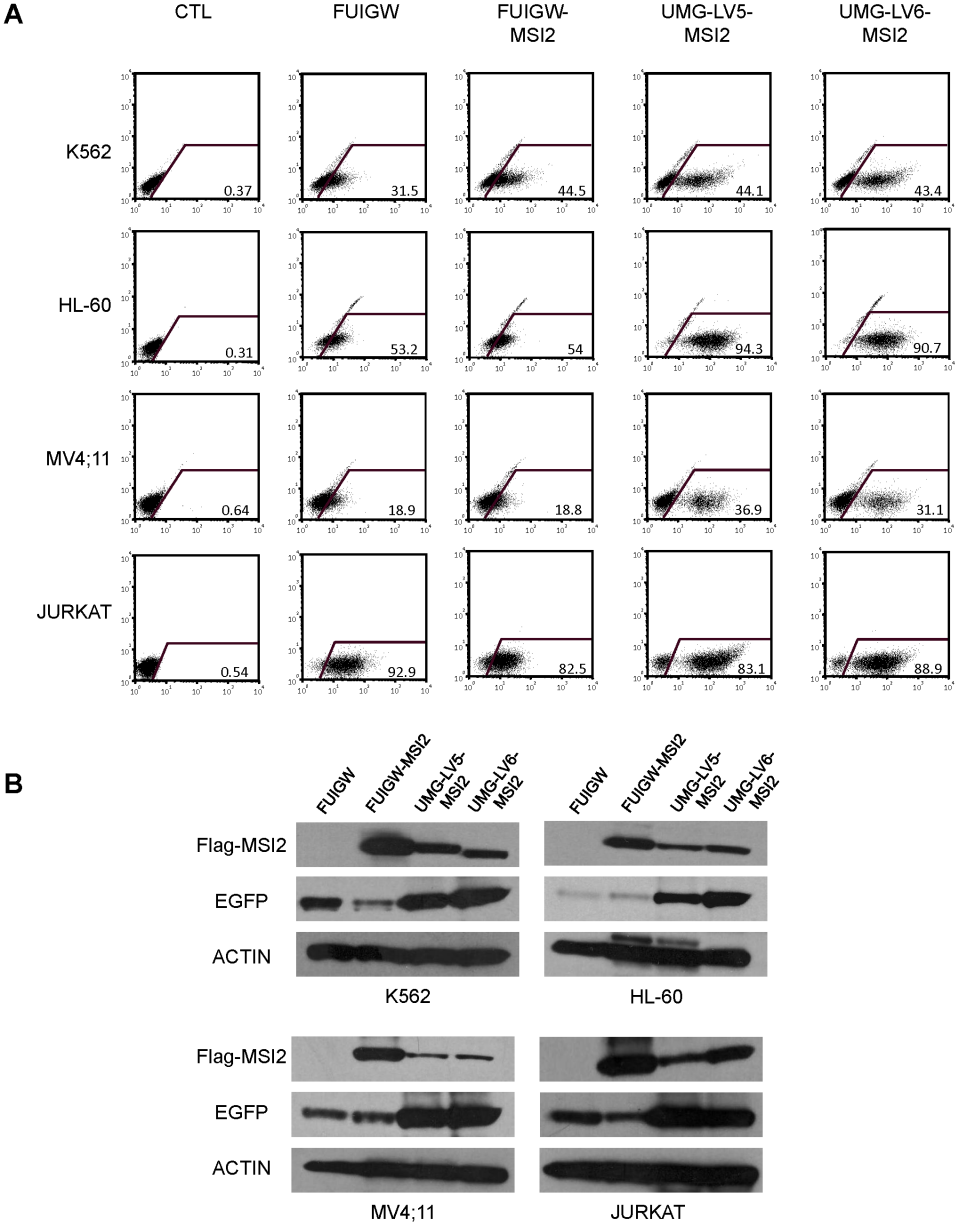
Comparison of the transduction efficiency of FUIGW, UMG-LV5 and UMG-LV6 carrying the MSI2 cDNA in human hematopoietic cell lines. The cell lines K562, HL-60, MV4;11 and Jurkat were infected with FUIGW, UMG-LV5 or UMG-LV6 viruses carrying 3xFLAG-MSI2 cDNA as a transgene. As a control, void FUIGW vector was used. (**A**) Flow-cytometric analysis of EGFP expression in cells exposed to the relevant vectors. The percentages of EGFP-positive cells are indicated. (**B**) Whole-cell extracts, prepared as described in materials and methods, were analyzed by Western blotting for FLAG-MSI2 and EGFP expression. Actin was used as a control for the amounts of extract loaded.

The results of these transduction experiments showed the same trend as the data obtained with the ZNF521 vectors: as illustrated in Fig. 3, both flow-cytometry assays (panel 3A) and Western blotting analyses (panel 3B) revealed a considerably higher expression of EGFP in the cells infected with UMG-LV5-MSI2 and UMG-LV6-MSI2 compared to FUIGW-MSI2, whereas the production of MSI2 was more abundant in the cells transduced with the latter vector (panel 3B).

### Assessment of the novel IRES-containing vector, UMG-LV-11

**Figure 4:**
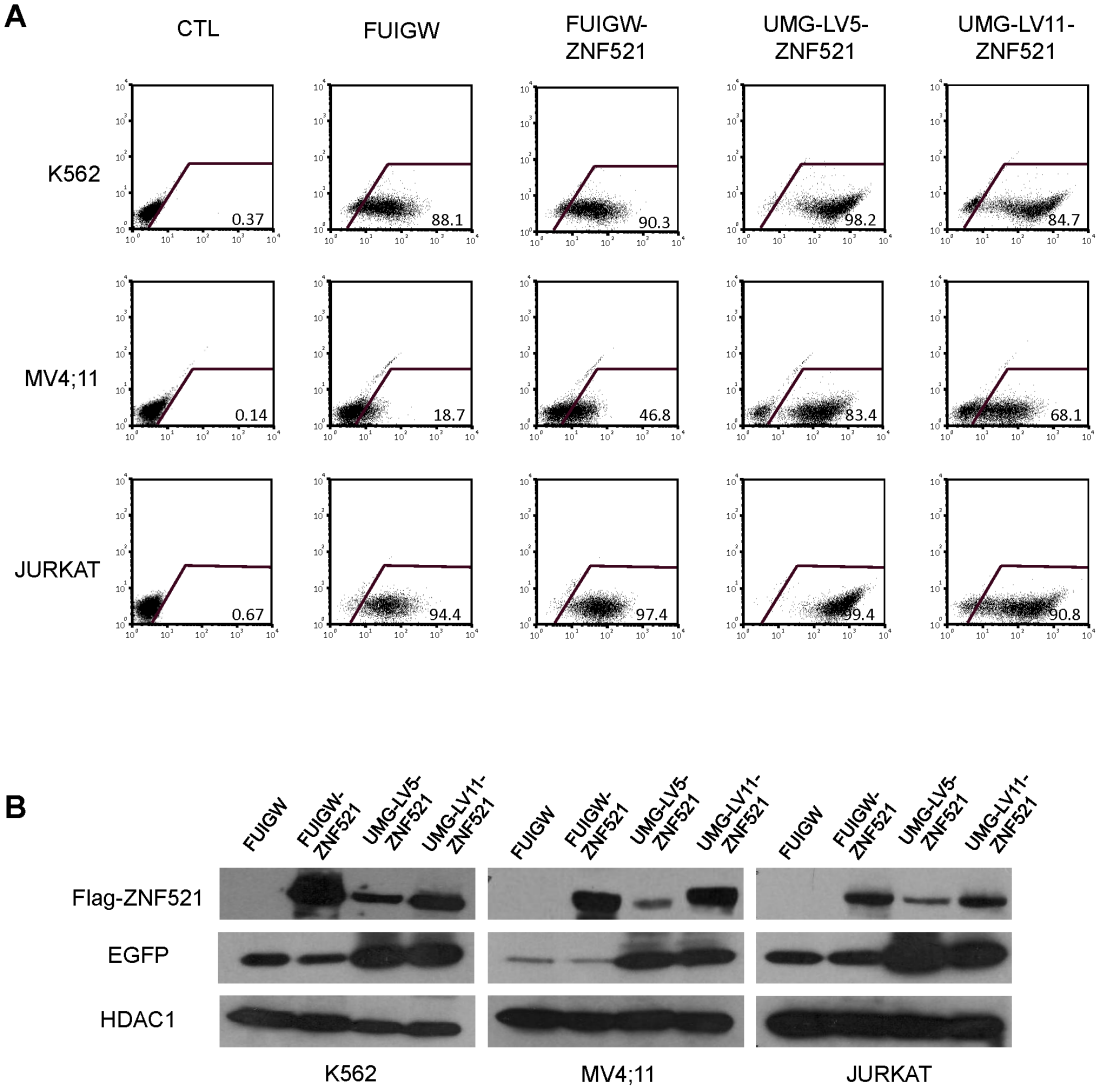
UMG-LV11 promotes efficient transgene- and reporter gene expression in human hematopoietic cell lines. The cell lines indicated were infected as detailed in materials and methods with FUIGW, UMG-LV5 or UMG-LV11 viruses carrying the cDNAs for 3xFLAG-ZNF521. As a control, void FUIGW vector was used. (**A**) Flow-cytometric analysis of EGFP expression in cells exposed to the relevant vectors. The percentages of EGFP-positive cells are indicated. (**B**) Nuclear and cytosolic extracts were analyzed by Western blotting for FLAG-ZNF521 and EGFP expression respectively. HDAC1 was used as a control for the amounts of extract loaded.

In bicistronic vectors, the translational efficiency is known to be variable in a manner that depends on the cell type and on the nature of the genes flanking the IRES element. In particular, it has been reported that while the cap-dependent translation of the upstream cDNA is relatively consistent, the IRES-dependent translation of the downstream gene is significantly influenced by the gene located upstream of the IRES [32]. We therefore asked whether inverting the positions of the cDNAs for the reporter protein and for the protein of interest, relative to the IRES sequence, may result in a more robust expression of both proteins. To this end, we constructed a new vector - named UMG-LV11 – where the EGFP cDNA was inserted upstream of the IRES, whereas the multiple cloning site for insertion of the transgene was downstream. The cDNA for ZNF521 was subcloned in UMG-LV11, and this vector was assayed on three haematopoietic cell lines in comparison with UMG-LV5-ZNF521. As shown in Fig. 4A, UMG-LV11-ZNF521 induced a strong expression of EGFP in all cell lines tested, fully comparable to that of UMG-LV5-ZNF521, although it displayed a slightly lower transduction efficiency.

Western blotting analyses (Fig. 4B) confirmed the strong expression of EGFP and highlighted a more abundant production of ZNF521 in all cells transduced with UMG-LV11-ZNF521 than in those infected with UMG-LV5-ZNF521, despite the reduced infection rate.

### Transduction efficiency of the UMG-LVs in CD34+ cells

**Figure 5:**
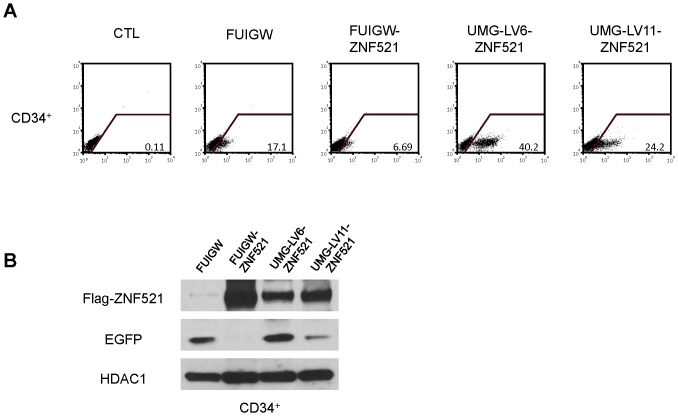
Efficiency of UMG-lenti vectors in the transduction of primary human CD34^+^ cells. CD34^+^ cells purified from cord blood were transduced with FUIGW, UMG-LV6 or UMG-LV11 viruses carrying the cDNAs for 3xFLAG-ZNF521 and EGFP. (**A**) FACS analysis of the transduced cells 5 days after transduction. The percentages of EGFP positive cells are indicated. (**B**) Western blotting analysis of FLAG-ZNF521 and EGFP expression was performed as described above on nuclear and cytosolic extracts. HDAC1 was used as a control for the amounts of extract loaded.

We next tested the efficiency of UMG-LVs in transducing primary hematopoietic stem and progenitor cells. Fig. 5 illustrates a representative experiment in which umbilical cord blood-derived CD34^+^ cells were subjected to two rounds of infection with FUIGW, FUIGW-ZNF521, UMG-LV6-ZNF521 and UMG-LV11-ZNF521. The highest percentage of EGFP^+^ cells, as well as the strongest mean fluorescence intensity were detected in the cultures transduced with UMG-LV6-ZNF521, followed by those exposed to UMG-LV11-ZNF521, while the EGFP^+^ cell fraction was negligible in the FUIGW-ZNF521-infected culture (Fig. 5A). These results were mirrored by the detection of corresponding levels of EGFP by Western blotting (Fig. 5B). Consistently with the findings obtained using hematopoietic cell lines, the amounts of 3xFLAG-ZNF521 produced by the FUIGW-ZNF521-infected cells were higher than those observed in the cells transduced with UMG-LV11-ZNF521 and UMG-LV6-ZNF521 (Fig. 5B).

**Figure 6:**
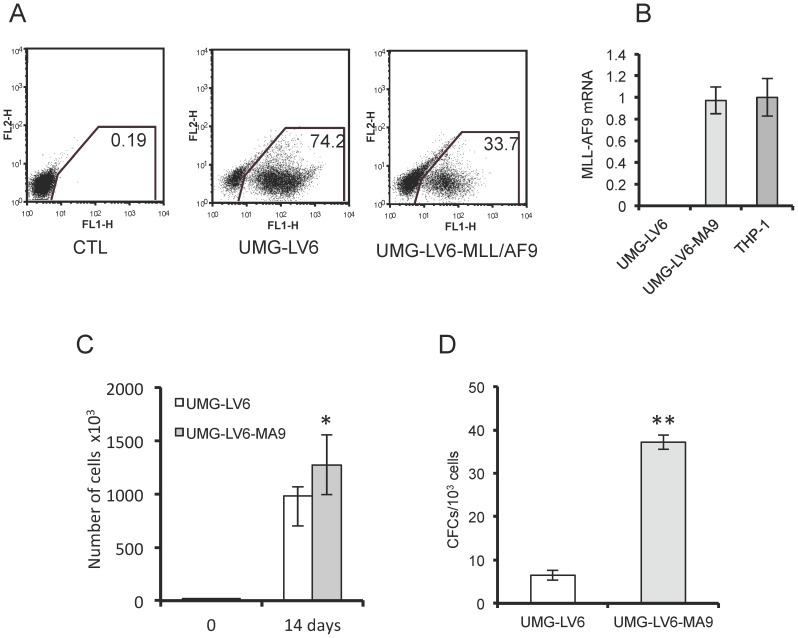
Transduction with UMG-LV6 carrying the MLL-AF9 fusion oncogene enhances the growth and clonogenicity of human CD34^+^ cells. CD34^+^ cells purified from cord blood were transduced with void UMG-LV6 vector or UMG-LV6 carrying the MLL-AF9 cDNA (UMG-LV6-MA). (**A**) FACS analysis of CD34^+^ cells 5 days after transduction. The percentages of EGFP positive cells are indicated. (**B**) Q-RT-PCR analysis of the expression of MLL-AF9 in CD34^+^ cells transduced with UMG-LV6-MA. The expression level was compared to that of the MLL-AF9-positive THP-1 cells, assumed as 1. (**C**) 1×10^4^ CD34^+^ cells transduced with void UMG-LV6 vector or with UMG-LV6-MA/well were plated in triplicate in 6-well plates in cytokine-driven cultures in the presence of 100 ng/ml of stem cell factor, FLT3 ligand and thrombopoietin. The culture medium was refreshed weekly, and the cell numbers were determined two weeks after plating. (**D**) The number of clonogenic progenitors in CD34^+^ cells transduced with void UMG-LV6 vector or with UMG-LV6-MA after two weeks of cytokine-driven culture was determined by clonogenic assays in methylcellulose as described in Matherials and Methods.

To confirm that the transgene expression ensured by UMG-LV6 - albeit lower than that obtained with FUIGW - was adequate to induce a detectable phenotype in primary HSPCs, we transduced human cord blood-derived CD34^+^ cells with an UMG-LV6 vector containing the cDNA encoding the fusion oncoprotein MLL-AF9, that has been shown capable of transforming human HSPCs in culture [8], [11]. The results of this experiment, summarized in Fig. 6, show that the enforced expression of MLL-AF9 driven by the UMG-LV6-MA9 vector (Fig. 6A, 6B) resulted in a proliferative advantage (Fig. 6C) and in a considerably higher frequency of immature, colony-forming cells (Fig. 6D) in transduced CD34^+^ cells.

### Relationship between transgene and EGFP expression in transduced K562 cells

**Figure 7:**
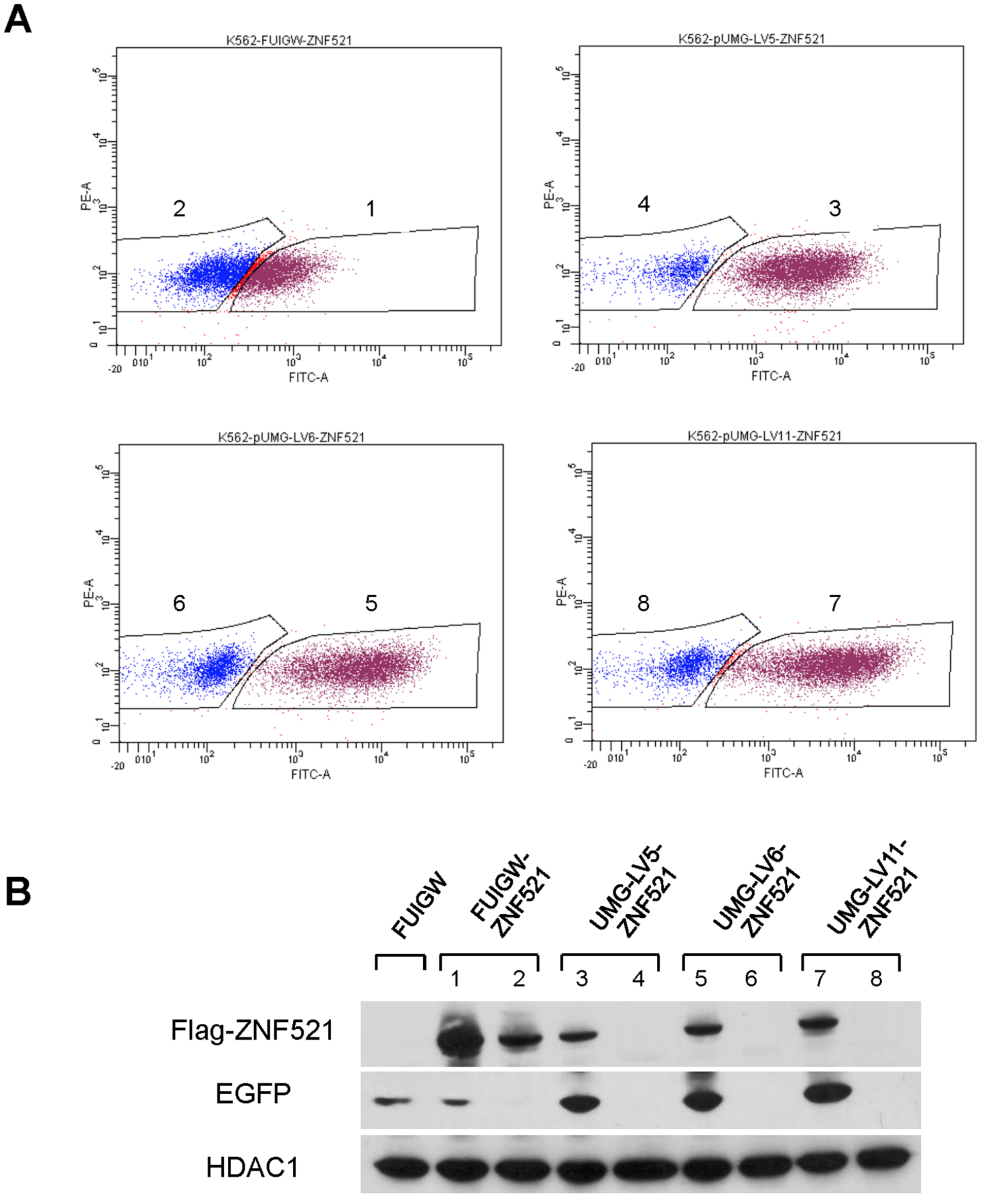
Transgene expression in transduced, sorted, EGFP^+^ and EGFP^−^ K562 cells. K562 cells were subjected to one round of transduction with the lentiviruses indicated in the figure. After 5 days the cells were sorted by FACSAriaIII based on EGFP expression (**A**), and the sorted EGFP-positive (gates 1, 3, 5, 7) and -negative (gates 2, 4, 6, 8) subpopulations were analyzed by western blotting for expression of 3xFLAG-ZNF521 and of EGFP (**B**). The purity of the sorted populations was subsequently evaluated by flow cytometry and is shown in S3 Figure.

The data reported above suggest that, owing to the low efficiency of the IRES sequence contained in FUIGW, a fraction of infected cells expressing the transgene produce very low levels of EGFP and therefore may escape flow-cytometric detection. To verify if this was the case, K562 cells were subjected to one round of infection with FUIGW-ZNF521, UMG-LV5-ZNF521, UMG-LV6-ZNF521 and UMG-LV11-ZNF521 and the EGFP-positive and negative cells were sorted by flow cytometry (Fig. 7A). The purity of the sorted cells was consistently ≥95% (S3 Figure). Nuclear extracts were prepared from the cell subpopulations thus isolated and assayed by Western blotting to measure the amounts of 3xFLAG-ZNF521. As shown in Fig. 7B, in the FUIGW-ZNF521-infected cultures both EGFP-positive and EGFP-negative cells displayed considerable transgene expression, compatible with an inadequate sorting of transduced cells owing to the presence of EGFP levels below the detection threshold. In contrast, in K562 cells infected with UMG-LV5-ZNF521, UMG-LV6-ZNF521 and UMG-LV11-ZNF521, the presence of 3xFLAG-ZNF521 was detected exclusively in the EGFP^+^ fraction indicating an efficient sorting of the transduced cells.

### Transduction of non-hematopoietic cells

**Figure 8:**
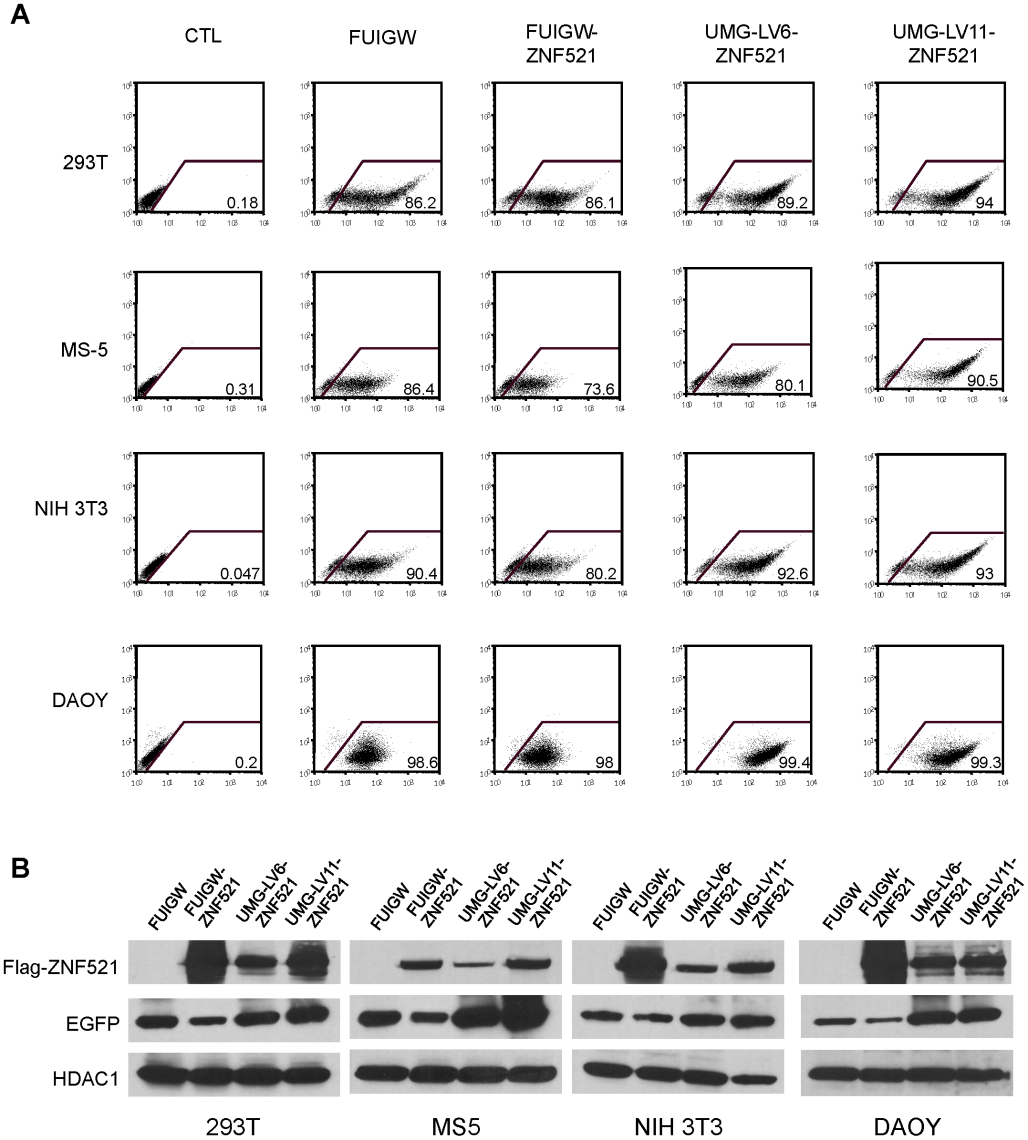
UMG-LVs efficiency in non hematopoietic cells. (**A**) Non-hematopoietic cell lines, HEK293T, MS-5, NIH-3T3 and DAOY, were transduced with FUIGW, FUIGW-ZNF521, UMG-LV6-ZNF521 and UMG-LV11-ZNF521 and analyzed by FACS to assess the percentage of EGFP positive cells. (**B**) Nuclear and cytosolic extracts were assayed with anti-FLAG and anti-EGFP antibodies as described above. HDAC1 was used as loading control.

The fragment of the WASP regulatory region used in the construction of the UMG-LV5 and UMG-LV6 vectors has been shown to direct the expression of the reporter gene in a tightly hematopoietic-specific manner [33] and therefore it would be expected to be functionally silent in other cell types. However, when used to infect human and murine non-hematopoietic cell lines derived from various tissues (human embryonyc kidney cells HEK293T, mouse mesenchymal stromal cells MS-5, mouse embryonic fibroblasts NIH3T3, and human medulloblastoma cells DAOY) UMG-LV6 promoted in all cases strong expression of both EGFP (Fig. 8A, 8B) and of transgene (Fig. 8B), comparable to those induced by UMG-LV11, suggesting that the functional interaction with the adjacent UBC promoter may overcome the tissue-specificity of the WASP regulatory element.

## Discussion

In this paper we report the construction and the validation of three novel lentiviral vectors for gene transfer that ensure efficient expression of transgenes and fluorescent reporter protein in cells of diverse hematopoietic cell lineages and, of particular relevance, in primary human CD34^+^ progenitor cells.

The first two vectors, designated UMG-LV5 and UMG-LV6, contain a bidirectional expression cassette where the transgene and the reporter protein are under the transcriptional control of two distinct promoters, that of the human Ubiquitin-C (UBC) gene and the minimal regulatory element of the Wiskott-Aldrich syndrome (WASP) gene, respectively. The choice of the latter was driven by its small size (170 nt, considerably shorter than the IRES element) and by its strong transcriptional activity in hematopoietic cells, with particular regard to stem and progenitor cells [33]–[34]. These vectors, that differ only for the orientation of the expression cassette, were tested on a panel of human myeloid and lymphoid cell lines in comparison with the IRES-based FUIGW vector [17]. In these assays, both UMG-LV5 and UMG-LV6 showed a substantially equivalent efficiency in promoting the expression of two distinct transgenes of different length (the cDNAs encoding 3xFLAG-tagged versions of the transcription co-factor ZNF521 and of the RNA-binding protein MSI2) and of EGFP. Cells infected with UMG-LV5 or UMG-LV6 resulted in dramatically more efficient expression of EGFP than those infected with FUIGW or other IRES-containing lentiviral vectors (such as pHIV-EF1α-ZNF521-IRES-EGFP, shown in S4 Figure), thus allowing the easy identification (Figs. 2, 3, 4, 5) and FACS sorting (Fig. 7) of transduced and non-transduced cell subsets. In contrast, the expression of the transgene product was consistently lower in cells infected with dual-promoter vectors compared to those transduced with FUIGW, probably due to some degree of interference between the two promoters. Transcriptional interference, the suppressive effect in *cis* between transcriptional units generally adjacent or overlapping, has been implicated in the regulation of genetic networks in prokaryotes as well as eukaryotes (reviewed in [35]). This phenomenon is frequently observed in retro- and lentiviral vectors carrying multiple promoters, and generally leads to the severe impairment of the transcriptional activity of at least one of the promoters [36]. In the case of UMG-LV5 and UMG-LV6, where the UBC and WASP promoter are positioned in back-to-back orientation, only a moderate decrease in transgene expression was detected; in previous attempts to generate dual-promoter constructs in which the same transcriptional units were organized in tandem, we invariably observed the almost complete repression of one of the two. Consistently, transduction of K562 cells with the commercial vector pCDH-CMV-ZNF521-EF1α-copGFP, where the transgene and the cDNA encoding the copGFP reporter protein are driven by tandem, potent ubiquitous promoters yielded in high levels of fluorescence but lower levels of transgene expression than those obtained with UMG-LV6-ZNF521 (S4 Figure).

We are currently exploring the possibility to insert insulator sequences between the two transcriptional units in order to abolish the possible interferences [37]. However, despite the relative decrease in the expression of the gene driven by the UBC promoter compared to FUIGW, considerable expression of the protein was achieved in all cases with UMG-LV5 and UMG-LV6, in particular in primary HSPCs (Fig. 5). Transduction of human CD34^+^ cells with UMG-LV6 carrying the MLL-AF9 oncogene provided proliferative advantage to the infected cells (Fig. 6) and ultimately resulted in their transformation [38] and in the acquisition of leukemogenic potential *in vivo* (Schuringa, personal communication). Thus, the levels of transgene expression induced by the dual-promoter constructs described are sufficient to generate a detectable phenotype in hematopoietic stem and progenitor cells. Furthermore, as illustrated in Fig. 8, the proximity of the potent ubiquitous UBC promoter appears to override the tissue-specificity of the WASP regulatory element, thereby ensuring strong GFP expression in a variety of non-hematopoietic cells of epithelial, mesenchymal and neural origin.

In a parallel approach, we sought to determine whether inserting the cDNA encoding the reporter protein upstream, and the transgene downstream of the IRES sequence may result in the efficient translation of both proteins. A new vector was therefore constructed, UMG-LV11, that contained the UBC-EGFP-IRES-transgene expression cassette. As documented in Figs. 4 and 5, the amounts of EGFP in all cells transduced with UMG-LV11-ZNF521 (including CD34^+^ cells) were considerably higher, and those of ZNF521 equivalent, to the levels observed in the cells infected by FUIGW-ZNF521.

In conclusion, the lentiviral vectors designated UMG Lenti, described in this paper, have proven highly efficient and reliable in infecting diverse cell types, including primary human hematopoietic progenitors, and in promoting robust expression of both transgene and fluorescent reporter protein, thereby enabling to easily monitor the transduction efficiency and to accurately sort the transduced cells. These vectors are amenable to further development, such as the incorporation of alternative reporter proteins, selectable genes, or multiple cloning sites for the insertion of a second transgene. As such they represent a panel of powerful reagents to enforce expression of genes of interest into a variety of primary immature cells. Although originally conceived with the hematopoietic system as a target, these vectors proved in fact able to transduce several non-hematopoietic cell types and they can be thus considered appropriate tools for more widespread applications.

## Supporting Information

S1 Figure Schematic map of the pUMG-LV5, pUMG-LV6 and pUMG-LV11 plasmids. Vector maps were generated using the SnapGene software (http://www.snapgene.com/). Unique restriction sites are indicated. A: pUMG-LV5; B: pUMG-LV6; C: pUMG-LV11.
(TIFF)

S2 Figure Comparison of the levels of EGFP expression in human hematopoietic cell lines transduced with FUIGW, FUIGW–ZNF521, UMG-LV5-ZNF521 and UMG-LV6-ZNF521. Flow-cytometry data are as in Fig. 2A, but the EGFP-positive cells have been separately analysed in distinct “low-EGFP” and “high-EGFP” gates based on the intensity of their fluorescence. The percentages of high- and low-EGFP-expressing cells are indicated in each panel.
(TIFF)

S3 Figure Flow-cytometric analysis of sorted EGFP^+^ and EGFP^−^ K562 cells. The experimental conditions are those described in Fig. 6. The purity of each sorted populations is indicated.
(TIFF)

S4 Figure Comparison of the GFP- and ZNF521 expression in K562 cells transduced with UMG-lenti vectors and commercially available IRES-containing or dual-promoter lentiviral vectors. K562 cells were subjected to one round of transduction with the vectors indicated, as described in Materials and Methods. Five days later the expression of GFP and of 3xFLAG-ZNF521 were analysed by flow-cytometry and western blotting respectively, as described in Materials and Methods. HDAC1 was used as internal control. The western blotting analysis of the GFP levels was not performed since the copGFP is not detected by the antibodies to GFP used in this paper. The percentages of GFP^+^ cells are indicated in each FACS plot.
(TIFF)

S5 Figure Original, full scans of the Western blots shown in Figs. 2B and 3B.
(TIFF)

S6 Figure Original, full scans of the Western blots shown in Figs. 4B, 5B, 7B and 8B.
(TIFF)
