# Supplementary figures and images for: UMG Lenti: Novel Lentiviral Vectors for Efficient Transgene- and Reporter Gene Expression in Human Early Hematopoietic Progenitors
Source: PLoS One. 2014 Dec 12;9(12):e114795. doi: 10.1371/journal.pone.0114795 (PMC4264771; doi:10.1371/journal.pone.0114795)

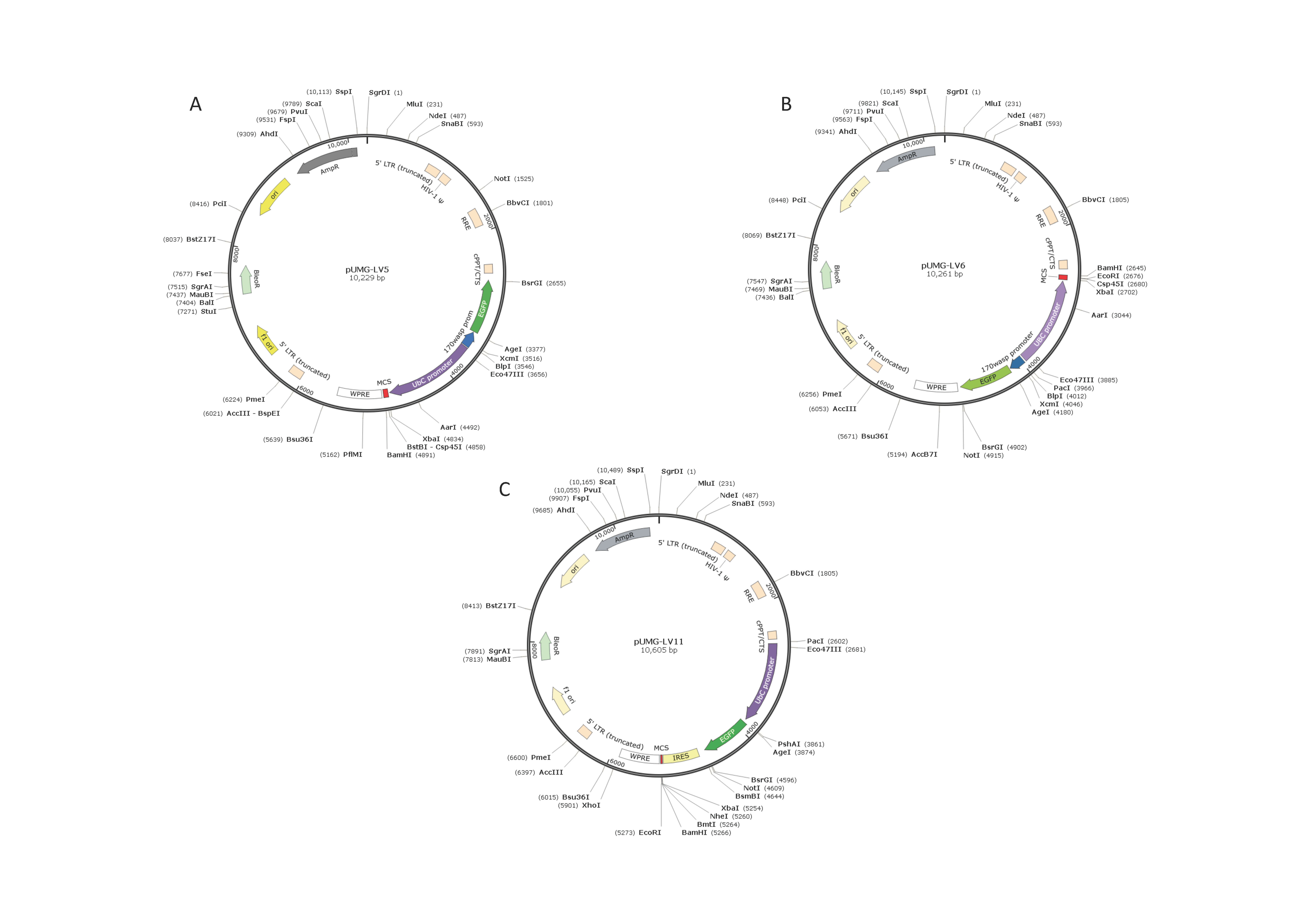

Supplement: S1 Figure — Schematic map of the pUMG-LV5, pUMG-LV6 and pUMG-LV11 plasmids. Vector maps were generated using the SnapGene software (http://www.snapgene.com/). Unique restriction sites are indicated. A: pUMG-LV5; B: pUMG-LV6; C: pUMG-LV11. (TIFF) [file pone.0114795.s001.tiff]

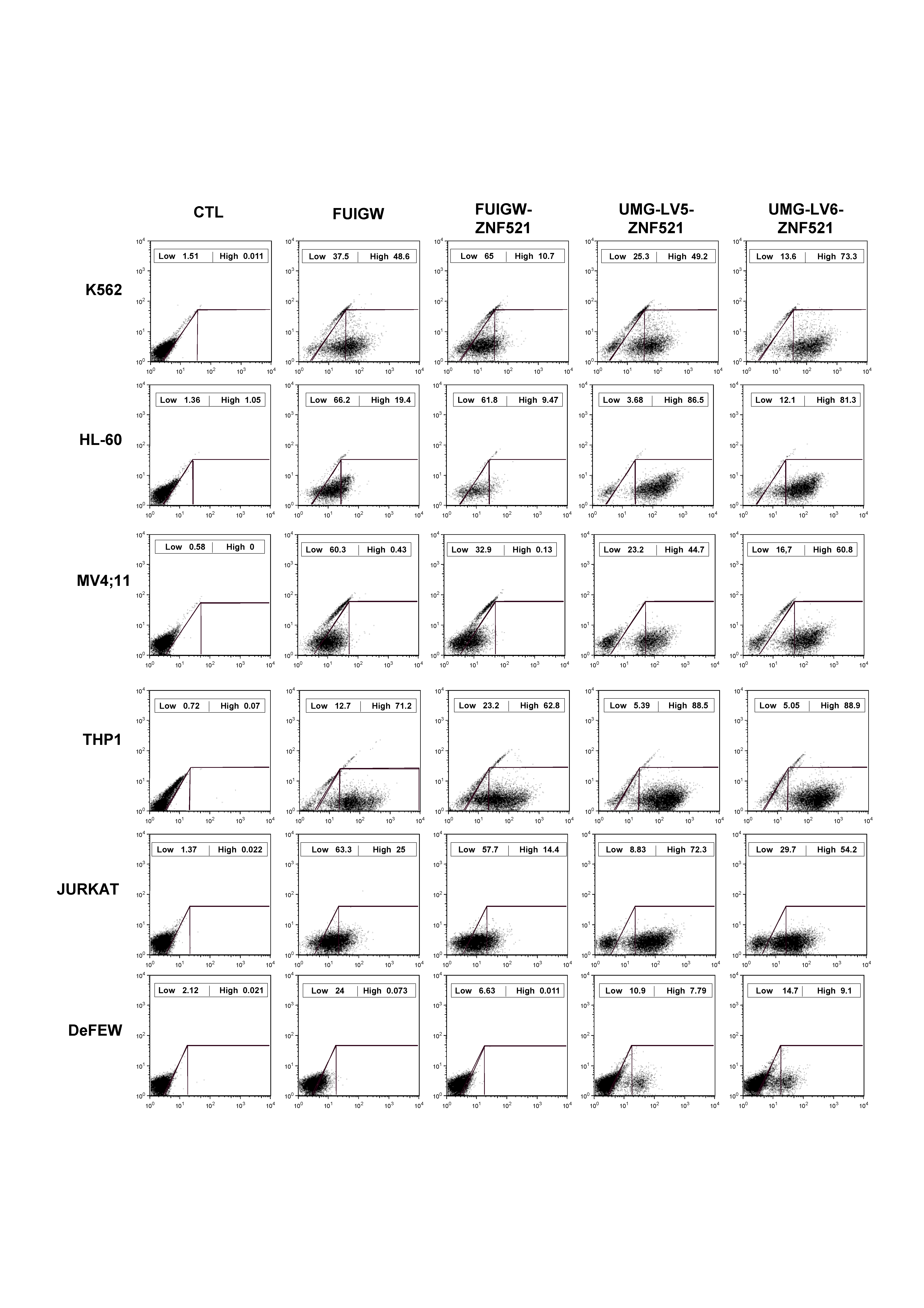

Supplement: S2 Figure — Comparison of the levels of EGFP expression in human hematopoietic cell lines transduced with FUIGW, FUIGW–ZNF521, UMG-LV5-ZNF521 and UMG-LV6-ZNF521. Flow-cytometry data are as in Fig. 2A, but the EGFP-positive cells have been separately analysed in distinct “low-EGFP” and “high-EGFP” gates based on the intensity of their fluorescence. The percentages of high- and low-EGFP-expressing cells are indicated in each panel. (TIFF) [file pone.0114795.s002.tiff]

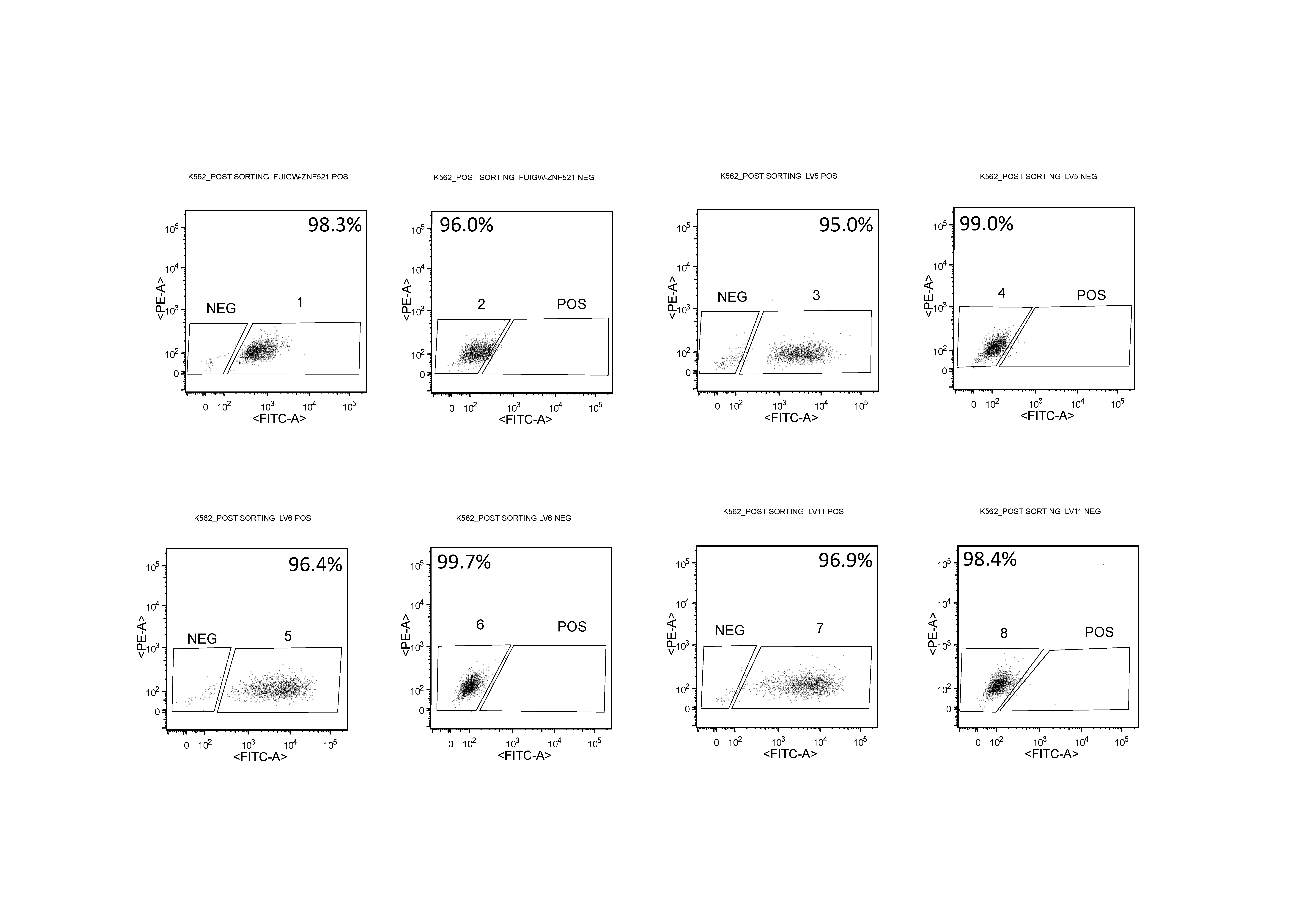

Supplement: S3 Figure — Flow-cytometric analysis of sorted EGFP+ and EGFP− K562 cells. The experimental conditions are those described in Fig. 6. The purity of each sorted populations is indicated. (TIFF) [file pone.0114795.s003.tiff]

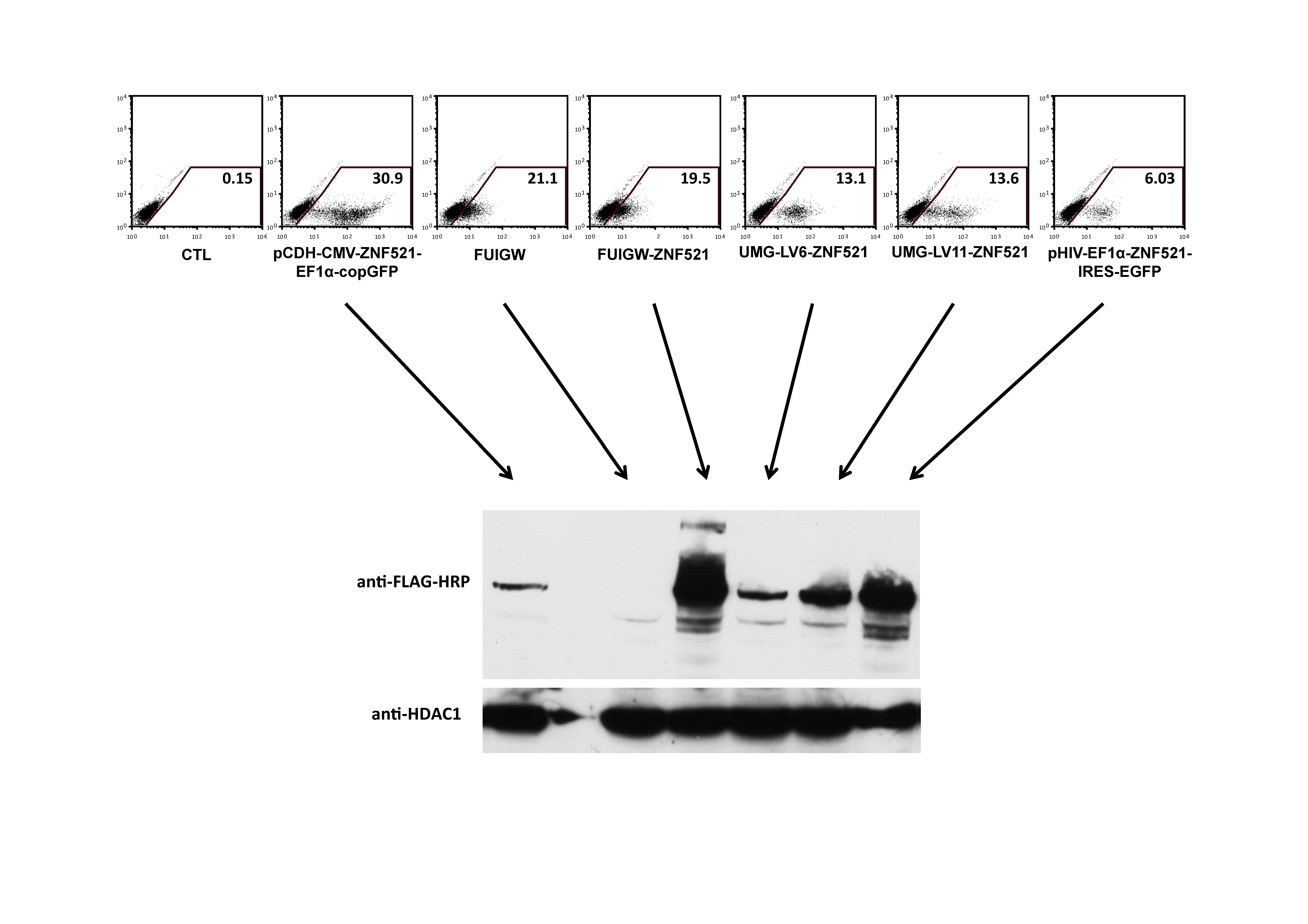

Supplement: S4 Figure — Comparison of the GFP- and ZNF521 expression in K562 cells transduced with UMG-lenti vectors and commercially available IRES-containing or dual-promoter lentiviral vectors. K562 cells were subjected to one round of transduction with the vectors indicated, as described in Materials and Methods. Five days later the expression of GFP and of 3xFLAG-ZNF521 were analysed by flow-cytometry and western blotting respectively, as described in Materials and Methods. HDAC1 was used as internal control. The western blotting analysis of the GFP levels was not performed since the copGFP is not detected by the antibodies to GFP used in this paper. The percentages of GFP+ cells are indicated in each FACS plot. (TIFF) [file pone.0114795.s004.tiff]

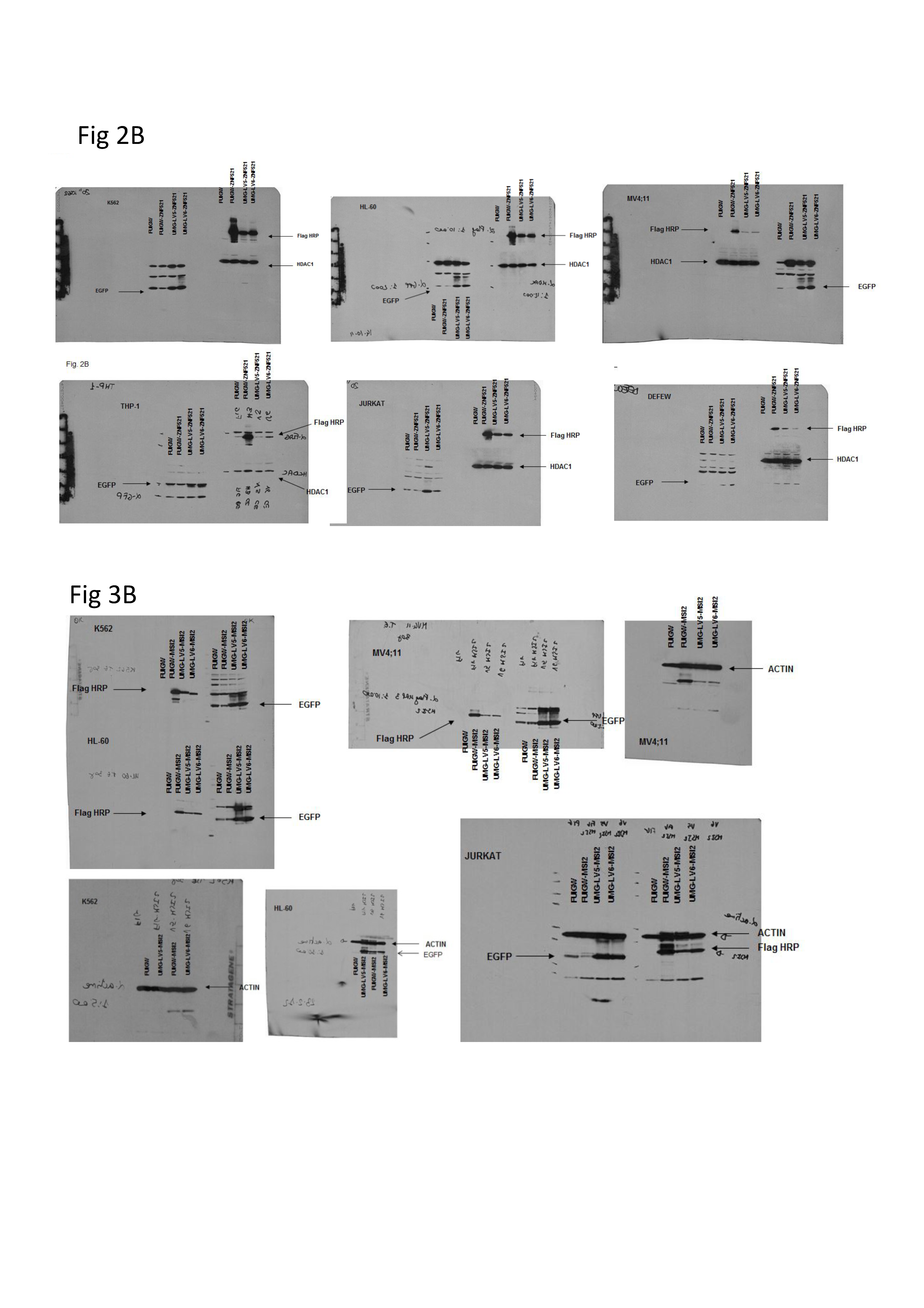

Supplement: S5 Figure — Original, full scans of the Western blots shown in Figs. 2B and 3B. (TIFF) [file pone.0114795.s005.tiff]

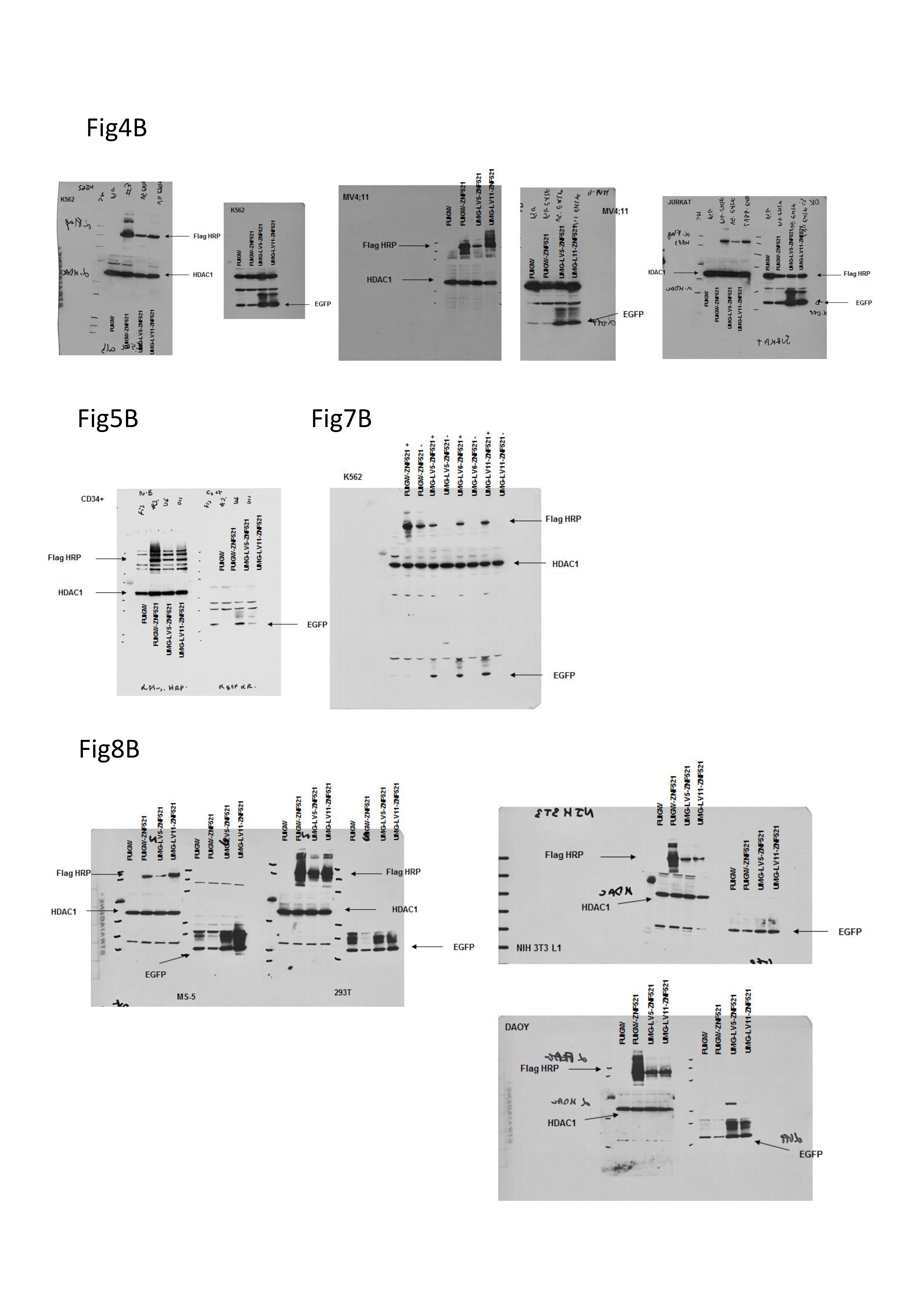

Supplement: S6 Figure — Original, full scans of the Western blots shown in Figs. 4B, 5B, 7B and 8B. (TIFF) [file pone.0114795.s006.tiff]
